# Risk Perceptions Regarding COVID-19 and Compliance with Protective Measures of Midwifery and Nursing Senior Students

**DOI:** 10.1590/1980-220X-REEUSP-2023-0303en

**Published:** 2024-03-08

**Authors:** Seval Cambaz Ulaş, Dilay Açıl, Damla Şahin Büyük, Seçil Köken Durgun, Fatma Uyar Açışlı

**Affiliations:** 1 Manisa Celal Bayar University, Faculty of Health Sciences, Midwifery Department/Manisa, Turkey.; 2 Manisa Celal Bayar University, Faculty of Health Sciences, Public Health Nursing Department/Manisa, Turkey.

**Keywords:** COVID-19, Health Risk, Vaccines, Security Measures, Health Occupations, Students, COVID-19, Risco à Saúde Humana, Vacinas, Medidas de Segurança, Ocupações em Saúde, Estudantes, COVID-19, Riesgo a la Salud, Vacunas, Medidas de Seguridad, Empleos en Salud, Estudiantes

## Abstract

**Objective:**

This study aims to examine the risk perceptions of midwifery and nursing senior students regarding COVID-19 and compliance with vaccination and protective measures.

**Method:**

This cross-sectional study was conducted in two academic years on senior midwifery and nursing students (n = 358). In the present study, the descriptive characteristics of the students and the COVID-19 risk perception scale were used.

**Results:**

The students’ COVID-19 Risk Perception Scale scores were at a moderate level and a similar level in both years of this study. More than 80% of the students were fully vaccinated, and the family history of COVID-19 was positive in approximately half of them. In the second year of the pandemic, they paid less attention to social distance and avoidance of being indoors.

**Conclusion:**

Although the COVID-19 risk perceptions of future health professional students remained at a similar level during the examined period, it was found that in the second year of the pandemic, they started to get used to the process and paid less attention to social protective measures.

## RESUMO

**Objetivo:**

Este estudo tem como objetivo examinar as percepções de risco de estudantes sêniores de obstetrícia e enfermagem em relação à COVID-19 e o cumprimento das medidas de vacinação e proteção.

**Método:**

Este estudo transversal foi realizado ao longo de dois anos acadêmicos com estudantes sêniores de obstetrícia e enfermagem (n = 358). No presente estudo, foram utilizadas as características descritivas dos estudantes e a escala de percepção de risco da COVID-19.

**Resultados:**

As pontuações dos estudantes na Escala de Percepção de Risco da COVID-19 situaram-se num nível moderado e semelhante em ambos os anos do estudo. Mais de 80% dos estudantes estavam totalmente vacinados, e aproximadamente metade deles tinha histórico familiar de COVID-19. No segundo ano da pandemia, prestaram menos atenção ao distanciamento social e aos hábitos de evitar permanência em espaços fechados.

**Conclusão:**

Embora as percepções de risco da COVID-19 dos futuros profissionais de saúde tenham permanecido em níveis semelhantes durante o período examinado, verificou-se que, no segundo ano da pandemia, começaram a acostumar-se com o processo e prestaram menos atenção às medidas de proteção social.

## RESUMEN

**Objetivo:**

Este estudio tiene como objetivo examinar las percepciones de riesgo de los estudiantes seniors de obstetricia y enfermería con respecto a COVID-19 y su cumplimiento con la vacunación y las medidas de protección.

**Método:**

Este estudio transversal se llevó a cabo en dos años académicos con estudiantes seniors de obstetricia y enfermería (n = 358). En el presente estudio, se utilizaron las características descriptivas de los estudiantes y la escala de percepción de riesgo de COVID-19.

**Resultados:**

Las puntuaciones en la Escala de Percepción de Riesgo de COVID-19 de los estudiantes estaban en un nivel moderado y similar en ambos años de este estudio. Más del 80% de los estudiantes estaban completamente vacunados y la historia familiar de COVID-19 fue positiva en aproximadamente la mitad de ellos. En el segundo año de la pandemia, prestaron menos atención a la distancia social y a evitar estar en interiores.

**Conclusión:**

Aunque las percepciones de riesgo de COVID-19 de los futuros profesionales de la salud se mantuvieron en un nivel similar durante el período examinado, se encontró que en el segundo año de la pandemia, empezaron a acostumbrarse al proceso y prestaron menos atención a las medidas de protección social.

## INTRODUCTION

The coronavirus disease (COVID-19) has affected approximately 665 million people worldwide and caused more than 6.7 million deaths. In our country, the total number of COVID-19 cases is over 17 million, and the total number of deaths is approximately 101.000^(1)^. Vaccination and protective measures are still important in the fight against COVID-19. The rate of getting at least 2 COVID-19 vaccines is 69.4% worldwide, while this rate is 68,3% in our country^(2)^. These rates are not at a satisfactory level to prevent the spread of the disease. However, effective control of the COVID-19 pandemic largely depends on the preventive measures taken by the community. Risk perception has been stated as an important factor affecting vital behaviors. High-risk perception is effective for maintaining protective behaviors^(3)^. COVID-19 risk perception of individuals in society may be affected by several factors, including previous exposure of themselves or their families to COVID-19, their general health state, personal values and beliefs, and trust in science, health professionals and the government^(3,4)^. As has been noticed during the pandemic process, healthcare professionals play a guiding role in protecting public health^(5)^. At the same time, society’s tendency towards correct preventive health behaviors is related to healthcare professionals’ awareness and management of risk perceptions^(6)^.

Attitudes and behaviors towards vaccination, which is the most important protective measure in protecting against Covid 19 disease, are affected by risk perception. However COVID-19 vaccination rates are not at the desired levels due to the risk perception of the population for the disease and hesitancy for vaccination^(7)^. This makes the control of the COVID-19 pandemic challenging; uncontrolled COVID-19 cases repeatedly infect others, and new variants appear and reduce the efficacy of vaccines. Society accepts the healthcare professionals who are at the forefront of the fight against the COVID-19 pandemic as the authority, and healthcare professionals are role models for them^(8)^. Thus, it is thought that the perceptions and behaviors of healthcare professionals regarding vaccination and protective measures affect the attitude of society toward COVID-19. Healthcare professionals’ acceptance of the COVID-19 vaccine was reported as 79.2% in Greece, 71.6% in Spain, 54% in Cyprus, 46.3% in Albania and 46.2% in Kosovo^(6)^. In another study, it was reported that only 45% of the faculty and student nurses participating in the study wanted to be vaccinated, and the reasons for not wanting to be vaccinated for COVID-19 were concerns about the safety and side effects of vaccines^(7)^. In a study on Egyptian nursing students, it was reported that the students were worried about the safety and side effects of vaccines. Only 47.4% agreed to be vaccinated, 35.8% were hesitant, and 16.8% refused^(8)^. In addition, a study examining the COVID-19 risk perception of Portuguese healthcare professionals reported that 54.9% of them supposed a high probability of contracting COVID-19, and 25% stated that their families were also at high risk of contracting COVID-19^(9)^. The findings of a study conducted in Turkey by Arslanca et al.^(10)^ reported the mean COVID-19 preventive behavior scores of health professionals as 85.6%. It was reported that only 66.9% of them wanted to be vaccinated.

On March 23, 2020, distance education was started in all primary and secondary schools and universities in Turkey due to the COVID-19 pandemic. Midwifery and nursing students’ ongoing hands-on training in health institutions and their involvement in the vaccination process in the 2020-2021 academic year might have affected their risk perceptions for COVID-19, their attitudes towards immunization, and their behaviors to maintain protective measures. It is thought that risk perception also affects healthcare professionals’ own health and professional processes. As a matter of fact, it has been determined that nursing students’ risk perceptions regarding Covid 19 directly affect their professional commitment^(5)^. Since it is the first pandemic experience of healthcare professionals candidates, it is thought that their perceptions of the process will affect their future professional experiences. In this context; this study aims to examine the risk perceptions of midwifery and nursing students regarding COVID-19, their compliance with vaccination and protective measures in the last two years during the pandemic.

## METHOD

### Design of Study

This is a cross-sectional study.

### Sample Definition

This study was conducted in the 2020–2021 and 2021–2022 academic years on the senior students of the Faculty of Health Sciences, Midwifery and Nursing Departments of a university in the Western part of Turkey. The total number of midwifery and nursing 4th-grade students is 260 annually (N:520). No sample selection method was used in this study. The sample of the present study consisted of 195 senior midwifery and nursing students in the 2020–2021 academic year, and 163 senior midwifery and nursing students in the 2021–2022 academic year who voluntarily agreed to participate in this study. The rate of participation in the study was 75.0% for the first year and 63.0% for the second year. The inclusion criterion was determined as being a senior midwifery or nursing student at the relevant faculty. Students who could not fully participate in hospital practices for any reason and who interrupted their education during the COVID-19 pandemic were excluded from the study.

### Data Collection

A descriptive data collection form and the COVID-19 Risk Perception Scale were used as measurement tools in this study.

*Descriptive data collection form:* It was prepared by the researchers in line with the literature. The data collection form consisted of 34 questions evaluating the sociodemographic characteristics of the students (12 questions), COVID-19 vaccination status (5 questions), and the status of taking protective measures for COVID-19 (17 questions).

*COVID-19 Risk Perception Scale:* The COVID-19 Risk Perception Scale (Yıldırım and Güler, 2020)^(11)^ was adapted from the SARS Risk Perception Scale (Brug et al., 2004)^(12)^. The five-point Likert-type scale consists of a total of 8 items and 2 sub-dimensions: emotional and cognitive. Both sub-dimensions of the scale consist of 4 items. A high score means a high-risk perception and a low score means a low-risk perception regarding COVID-19^(11)^. Cronbach’s alpha internal consistency coefficient was calculated as 0.73 for the cognitive dimension of the scale and 0.88 for the emotional dimension. Kaiser Mayer Olkin (KMO) value of the scale is 0.80. During our study, the reliability and validity of the COVID-19 Risk Perception Scale were re-measured. The Cronbach’s alpha value of the cognitive sub-dimension of the scale is 0.73 and the Cronbach’s alpha value of its emotional sub-dimension is 0.90 for this study. The Kaiser–Meyer–Olkin Measure of Sampling Adequacy was acceptable (KMO = 0.754) and the p-value of Bartlett test of sphericity was <0.001.

The data of the study were collected in two academic years. The data were gathered in March in the spring semester of the 2020–2021 academic year in the first year of the pandemic and in September in the fall semester of the 2021–2022 academic year in the second year of the pandemic. The data were collected by face-to-face interview method, under observation, in approximately 20 minutes.

### Data Analysis and Treatment

All data were analyzed with SPSS (Statistical Package for Social Sciences), Version 20.0 software (SPSS, Inc., Chicago, IL, USA). The conformity of the COVID-19 Risk Perception Scale scores to normal distribution was analyzed by the skewness (± 1.96) and kurtosis (±1.96) values, Komogorov-Smirnov test. As the data were not normally distributed, Mann-Whitney U and Kruskal-Wallis tests were used to relate categorical independent variables to the COVID-19 Risk Perception Scale, the main dependent variable of the study. Categorical variables were presented as frequencies and percentages, and continuous variables were presented as medians and interquartile ranges. COVID-19 Risk Perception Scale remeasured the reliability and validity analyses (KMO, Barlett test and Cronbach apha).

### Ethical Aspects

This study was approved by Manisa Celal Bayar University Faculty of Medicine Health Sciences Ethics Committee (No: 20.478.486-993). The students were informed about this study, and written and verbal permissions were obtained.

## RESULTS

In the first year of the pandemic, 62% of the students were 22 years old or younger, 86.2% of them were female, about 64% of them were nursing students, and 47% were staying with their families. In the second year of the pandemic, 85% of the students were 22 years old or younger, 83.4% were female, about 64% were nursing students, and 54% stayed in the dormitory.

Of the students, 14.4% had COVID-19 In the first year, and 11.7% s had it in the second year of the pandemic, and it was determined that the most common symptom was fever. The COVID-19 vaccination rates of the students in the first and second years of the pandemic were 92.8% and 100.0%, respectively. The rates of fully vaccinated students were 81.0% and 87.7%. In the first year of the pandemic, almost all of the students were vaccinated with Sinovac. It was determined that the majority of them were vaccinated with BioNTech in the second year. In both years, approximately half of the students experienced a post-vaccine reaction, and regional pain was the most common one. About half of the students’ families had COVID-19. The COVID-19-related death rate in their families was 9.7% in the first year of the pandemic, but this rate decreased to 6.1% in the second year (Table 1).

**Table 1 –:** COVID-19 status and vaccination characteristics of the students – Manisa, Turkey, 2022.

		1st year		2nd year	
		n	%	n	%
**Had COVID-19**	Yes	28	14.4	19	11.7
	No	167	85.6	144	88.3
**Signs**	Fever	12	6.2	9	5.5
	Cough	8	4.1	7	4.3
	Headache	5	2.6	6	3.7
	Throat pain	1	0.5	3	1.8
	Rhinorrhea	1	0.5	1	0.6
	Malaise-fatigue	9	4.6	5	3.1
	Dyspnea	4	2.1	1	0.6
	Waist-back pain	0	0.0	3	1.8
	Nausea-vomiting	3	1.5	0	0.0
	Loss of taste/smell	9	4.6	5	3.1
	Muscle-joint pain	9	4.6	6	3.7
	Palpitation	1	0.5	0	0.0
**COVID-19 vaccination**	Yes	181	92.8	163	100.0
	No	14	7.2	0	0.0
**Vaccination status**	Fully vaccinated	158	81.0	143	87.7
	Not vaccinated/not fully vaccinated	37	19.0	20	12.3
**Reason for not getting vaccinated**	(S)he thinks he already has antibodies	8	2.2	0	0.0
	Distrust	6	1.7	0	0.0
**Vaccine**	Sinovac	141	72.3	12	7.4
	BioNTech	17	8.7	134	82.2
	Sinovac + BioNTech	13	6.7	7	4.3
**Experiencing a post-vaccination reaction**	Yes	80	41.0	86	52.8
	No	102	52.3	74	45.4
**Post-vaccination reaction**	Regional pain	79	40.5	91	55.8
	Regional swelling	11	5.6	15	9.2
	Regional erythema	5	2.6	10	6.1
	Mild fever	10	5.1	30	18.4
	Chills	10	5.1	22	13.5
	Diarrhea	1	0.5	0	0.0
	Muscle-joint pain	18	9.2	34	20.9
	Nausea-vomiting	10	5.1	12	7.4
	Headache	29	14.9	32	19.6
	Malaise-fatigue	52	26.7	60	36.8
	Rhinorrhea	1	0.5	0	0.0
**Having COVID-19 after vaccination (n = 344)**	Yes	3	1.7	5	3.1
	No	178	98.3	158	96.9
**Family history of COVID-19**	Yes	87	44.6	75	46.0
	No	108	55.4	88	54.0
**The degree of proximity of the relative who had COVID-19**	First-degree relative	33	16.9	44	27.0
	Second-degree relative	52	26.7	35	21.5
	Other	33	16.9	22	13.5
**COVID-19-related deaths in the family**	Yes	19	9.7	10	6.1
	No	176	90.3	153	93.9

When the COVID-19 protective measures of the students were examined in the first and second years of the pandemic, the findings showed that 94.1% and 92.6% of them washed their hands frequently, 84.9% and 81.6% of them always wore masks outside the home, 86.2% and 82.8% of them paid attention to the measures to protect themselves, their teammates and the patients while working in the clinic (Table 2).

**Table 2 –:** Protective measures taken and the perception of COVID-19 among the students – Manisa, Turkey, 2022.

		1st year		2nd year	
		n	%	n	%
**Protective measures taken for COVID-19**	I wash my hands frequently.	185	94.9	151	92.6
	I always wear my mask when I’m out of the house.	171	87.7	133	81.6
	While working in the clinic, I pay attention to the precautions to protect myself, my teammates and my patients.	168	86.2	135	82.8
	I follow the social distancing rules.	155	79.5	88	54.0
	I avoid being in indoors.	136	69.7	62	38.0
	I always have hand sanitizer/cologne with me when I go out.	135	69.2	90	55.2
	I change my mask when necessary and after using it for a maximum of four hours.	131	67.2	73	44.8
	I wear double masks.	119	61.0	41	25.2
	I do not meet my friends indoors.	32	16.4	5	3.1
	I don’t take public transport.	20	10.3	5	3.1
	I don’t eat or drink anything outside.	10	5.1	0	0.0
**Influence of clinical practice on the perception of COVID-19**	I have better learned about COVID-19 prevention measures.	154	79.0	108	66.3
	Continuing to practice during the COVID-19 pandemic gave me relief before I started my professional life.	142	72.8	93	57.1
	I felt lucky to have been allowed to get the COVID-19 vaccine.	140	71.8	43	26.4
	My perception of the seriousness towards the COVID-19 infection has increased.	19	9.7	25	15.3
	I felt bad about having to get the COVID-19 vaccine.	18	9.2	10	6.1
	Continuing to practice during the COVID-19 pandemic worried me before I started my career.	7	3.6	17	10.4
**Total**		195	100.0	163	100.0

The cognitive dimension scores of female students in the first year of the COVID-19 pandemic, the emotional dimension scores of female students in the second year, and the risk perception scores of female students in both years were higher (p < 0.05). In addition, in the first year, midwifery students had higher COVID-19 risk perception scores compared to nursing students, and the difference was statistically significant (p < 0.05). In the second year, both the emotional dimension and the total COVID-19 risk perception scores were higher in students who did not have a family history of COVID-19 when compared to the ones with a family history of COVID-19 (p < 0.05) (Table 3).

**Table 3 –:** Comparison of the students’ perception of COVID-19 risk and influencing factors – Manisa, Turkey, 2022.

Descriptive characteristic		COVID-19 Risk Perception Scale, Cognitive Dimension		COVID-19 Risk Perception Scale, Emotional Dimension		COVID-19 Risk Perception Scale, Total Score	
		1st year 9.11 ± 3.13 (4.00–18.00)	2nd year 10.62 ± 4.00 (4.00–20.00)	1st year 12.27 ± 5.27 (4.00–20.00)	2nd year 13.21 ± 5.25 (4.00–20.00)	1st year 21.37 ± 7.01 (8.00–36.00)	2nd year 23.83 ± 8.20 (8.00–40.00)
		Median (IQR)	Median (IQR)	Median (IQR)	Median (IQR)	Median (IQR)	Median (IQR)
**Age group**	≥22	8.50 (2.00)	10.00 (5.00)	12.00 (10.25)	14.00 (9.00)	21.00 (11.00)	24.00 (13.00)
	≤23	9.00 (4.50)	12.00 (3.00)	12.00 (7.00)	14.00 (10.25)	22.00 (9.00)	26.00 (10.25)
	Statistical Test*	z = –0.071 p = 0.943	z = –1.292 p = 0.196	z = –0.540 p = 0.589	z = –0.148 p = 0.882	z = –0.413 p = 0.679	z = –0.743 p = 0.458
**Gender**	Female	9.00 (4.75)	11.00 (6.00)	12.00 (10.00)	14.00 (8.00)	22.00 (11.00)	25.00 (12.00)
	Male	7.00 (6.00)	10.00 (3.00)	11.00 (8.00)	9.00 (9.00)	17.00 (10.00)	20.00 (9.00)
	Statistical Test*	**z = –3.317** **p = 0.001**	z = –1.876 p = 0.061	z = –1.741 p = 0.082	**z = –3.175** **p = 0.001**	**z = –2.727** **p = 0.006**	**z = –2.998** **p = 0.003**
**Department**	Midwifery	10.00 (5.00)	9.00 (6.50)	12.00 (11.00)	13.50 (12.25)	22.00 (12.00)	22.00 (16.75)
	Nursery	8.00 (4.00)	11.00 (4.50)	12.00 (8.00)	14.00 (7.00)	21.50 (10.00)	25.00 (9.00)
	Statistical Test*	**z = –2.262** **p = 0.024**	z = –0.956 p = 0.339	z = –.290 p = 0.772	z = –0.795 p = 0.426	z = –0.663 p = 0.508	z = –1.017 p = .309
**Stays in**	Family House	9.00 (4.00)	11.00 (5.50)	12.50 (10.75)	14.00 (8.75)	22.00 (11.00)	24.00 (12.00)
	Student House	8.00 (7.00)	10.50 (5.00)	12.00 (7.50)	16.00 (11.50)	20.00 (8.00)	25.50 (13.50)
	Dorm	9.00 (5.00)	10.00 (6.00)	11.50 (8.25)	14.00 (9.00)	21.00 (13.25)	24.00 (11.00)
	Statistical Test*	x^2^ = 1.302 p = 0.521	x^2^ = 0.197 p = 0.906	x^2^ = 1.473 p = 0.479	x^2^ = 1.995 p = 0.369	x^2^ = 0.163 p = 0.922	x^2^ = 0.557 p = 0.757
**Family history of COVID-19**	Yes	9.00 (4.00)	10.00 (6.00)	12.00 (6.00)	12.00 (9.00)	22.00 (12.00)	22.00 (14.00)
	No	9.00 (4.00)	11.00 (5.75)	12.00 (9.75)	15.00 (6.00)	21.00 (10.75)	25.00 (11.00)
	Statistical Test*	z = –0.271 p = 0.787	z = –1.503 p = 0.133	z = –1.516 p = 0.130	**z = –2.878** **p = 0.004**	z = –0.985 p = 0.325	**z = –2.509** **p = 0.012**
**Deaths in the family**	Yes	8.00 (5.00)	11.00 (9.00)	10.00 (6.00)	16.00 (15.25)	18.00 (8.00)	26.00 (4.25)
	No	9.00 (2.00)	11.00 (5.00)	12.00 (9.00)	14.00 (9.00)	22.00 (10.75)	24.00 (11.00)
	Statistical Test*	z = –1.064 p = 0.287	z = 0.000 p = 1.000	z = –0.640 p = 0.522	z = –0.555 p = 0.579	z = –1.041 p = 0.298	z = –0.235 p = 0.814
**Personal history of COVID-19**	Yes	8.50 (3.75)	10.00 (4.00)	13.50 (9.00)	14.00 (8.00)	22.50 (8.25)	25.00 (12.00)
	No	9.00 (5.00)	11.00 (5.00)	12.00 (9.00)	14.00 (8.75)	21.00 (10.00)	24.00 (11.75)
	Statistical Test*	z = –0.520 p = 0.603	z = –0.231 p = 0.817	z = –1.636 p = 0.102	z = –0.755 p = 0.450	z = –1.025 p = 0.305	z = –0.458 p = 0.647
**Vaccination**	Fully vaccinated	10.00 (5.00)	11.00 (5.00)	13.00 (9.00)	14.00 (10.00)	23.00 (12.00)	26.00 (13.00)
	Not fully vaccinated/not vaccinated	10.00 (5.50)	10.50 (5.50)	12.00 (9.00)	16.00 (7.50)	22.00 (10.00)	24.00 (9.75)
	Statistical Test*	z = –0.031 p = 0.975	z = –0.195 p = 0.845	z = –1.066 p = 0.287	z = –1.414 p = 0.157	z = –0.808 p = 0.419	z = –1.045 p = 0.296

* Mann-Whitney U test

** Kruskal-Wallis Test.

When the COVID-19 risk perception scores of students in the first and second years of the COVID-19 pandemic were compared with the measures they took to protect themselves from COVID-19, the total COVID-19 risk perception score was significantly higher in those who paid attention to social distance rules in the first year of the pandemic, those who avoided being indoors, those who always had hand sanitizer/cologne with them when they went out, those who used double masks, those who said that they did not learn about protection measures, and those who thought that they felt lucky because they were given the opportunity to have the COVID-19 vaccine. In the second year of the pandemic, the total COVID-19 risk perception score was significantly higher in those who always had hand sanitizer/cologne with them when they went out and those who wore double masks (p < 0.05) (Table 4).

**Table 4 –:** Comparison of the measures taken by the students to protect themselves from COVID-19, their perception of COVID-19, and the total scale scores – Manisa, Turkey, 2022.

Protective measures for COVID-19	COVID-19 Risk Perception Scale Total Score		
		1st year	2nd year
		Median (IQR)	Median (IQR)
I wash my hands frequently.	Yes	22,00 (10,00)	25,00 (12,00)
	No	17,00 (17,75)	20,00 (7,25)
	Statistical Test*	z = –0,950, p = 0,342	z = –0,878, p = 0,380
I always wear my mask when I’m out of the house.	Yes	22,00 (9,00)	25,00 (11,00)
	No	18,00 (11,75)	23,00 (12,75)
	Statistical Test*	z=-1,755, p = 0,079	z = –0,426, p= 0,670
While working in the clinic, I pay attention to the precautions to protect myself, my teammates, and my patients.	Yes	21,00 (10,00)	24,00 (12,00)
	No	24,00 (10,00)	25,00 (12,75)
	Statistical Test*	z = –1,793, p = 0,073	z = –0,225, p = 0,822
I follow the social distancing rules.	Yes	22,00 (11,50)	24,00 (11,00)
	No	19,00 (11,00)	24,00 (12,00)
	Statistical Test*	z = –2,015, **p = 0,044**	z = –0,622, p = 0,534
I always have hand sanitizer/cologne with me when I go out.	Yes	23,00 (10,00)	25,50 (11,00)
	No	18,00 (11,75)	22,00 (11,50)
	Statistical Test*	z = –2,757, **p =0,006**	z = –2,452, **p = 0,014**
I change my mask when necessary and after using it for a maximum of four hours.	Yes	22,00 (11,00)	24,00 (11,00)
	No	19,50 (11,50)	24,50 (12,25)
	Statistical Test*	z = –1,110, p = 0,267	z = –0,179, p = 0,858
I avoid being in indoors.	Yes	23,00 (10,00)	26,00 (12,25)
	No	18,00 (12,00)	24,00 (12,00)
	Statistical Test*	z = –2,171, **p = 0,030**	z = –1,695, p = 0,090
I wear double masks.	Yes	23,00 (111,00)	27,00 (11,00)
	No	19,00 (10,75)	23,00 (13,00)
	Statistical Test*	z = –2,462, **p = 0,014**	z = –3,172, **p = 0,002**
I have better learned about COVID-19 prevention measures.	Yes	20,50 (10,25)	24,50 (12,50)
	No	25,00 (13,00)	24,00 (11,00)
	Statistical Test*	z = –3,242, **p = 0,001**	z = –0,223, p = 0,823
Continuing to practice during the COVID-19 pandemic gave me relief before I started my professional life.	Yes	22,00 (10,00)	25,00 (11,00)
	No	23,00 (12,50)	23,00 (12,00)
	Statistical Test*	z = –0,267, p = 0,790	z = –0,114, p = 0,909
I felt lucky to have been given the opportunity to get the COVID-19 vaccine.	Yes	23,00 (10,00)	25,00 (10,00)
	No	19,00 (11,00)	24,00 (11,75)
	Statistical Test*	z = –2,306, **p = 0,021**	z = –0,296, p = 0,767

* Mann-Whitney U test.

## DISCUSSION

Risk refers to both the likelihood of harm and the seriousness of the harmful consequences if they occur. At least two dimensions of risk perception have been defined in the literature: the cognitive component and the sensory/emotional component^(13)^. A major shortcoming of current research on risk perception is focusing on one point in time and not assessing change over time^(14)^. This research, on the other hand, aims to evaluate the risk perception over a wider period by determining the risk perceptions of senior midwifery and nursing students towards COVID-19 in two years. In the present study, the risk perception of the students about COVID-19 was at a moderate level, and the risk perception of the students about COVID-19 was at a similar level in another study conducted on medical students in Turkey^(15)^. However, a study conducted on medical students in Iran, a study on dentistry students in Malaysia, and studies on young Italian adults and university students in Ethiopia found high-risk perceptions for COVID-19^(16)^. The variability of risk perception for COVID-19 in the aforementioned studies may be explained by cultural differences, the use of different assessment tools, and assessment at different times of the pandemic. When the change in risk perception over time was evaluated, the findings showed that students’ cognitive, emotional and total risk perceptions were at a moderate level in the first year of the pandemic, and slightly above the medium level in the second year. In some studies, it has been shown that risk perception increases over time^(14)^. It should be noted that these studies are insufficient in number.

In this study, the risk perception for COVID-19 was statistically significantly higher in female midwifery students who did not have a family history of COVID-19. In another study conducted at the same university, the mean total scores of the COVID-19 fear scale were statistically significantly higher in female midwifery students than in male students (p < 0.01)^(17)^. Female students in the medical and dentistry department of a university in Malaysia had higher risk perceptions than male students^(18)^. Given that all students in the midwifery department are women, also supports this finding. Consistent with a study conducted in China, college students reported worrying about their older family members^(19)^. In the study, it was determined that the effects of demographic variables on risk perception were limited, in line with the literature^(20)^.

In the first year of the pandemic, the rate of unvaccinated students for COVID-19 was 7.2%. Unvaccinated students stated the reasons for not getting vaccinated as believing they had antibodies and distrust in the vaccine. In another study, 16.7% of nursing students stated that they did not want to be vaccinated, and all of these students were concerned about the safety of the vaccine and did not trust the vaccine^(21)^. In the study by Salmon et al.^(22)^, it was reported that the rate of individuals who never thought of having vaccinated was 10%. In a study conducted in the United Kingdom, Paul et al.^(23)^ reported that 16% of participants had a high level of distrust of the COVID-19 vaccine. In the study conducted by Salali and Uysal^(24)^, it was stated that those who were worried about COVID-19 were more likely to get vaccinated. In this study, the senior students were being educated in the health field and carried out their applied courses in the hospital. These might have caused higher risk perceptions for the pandemic and increased their desire to be vaccinated immediately. Perception of risk played a key role in the desire to get vaccinated and increased the desire to get a COVID-19 vaccine even in more hesitant participants^(7)^. The findings showed that the students with a high-risk perception felt lucky to have the COVID-19 vaccine in the first period of the pandemic, and this was statistically significant (Table 4). It can be said that this finding is in line with the literature. The rapidity of the production, release and application processes of COVID-19 vaccines worldwide might have caused universal hesitation^(11)^. From this point of view, it can be emphasized that midwifery and nursing students have low perceptions of barriers to vaccination.

In this study, it was determined that the students complied with most of the protective measures, such as paying attention to social distancing, wearing masks indoors and frequent hand washing in the first year of the pandemic. In the second year of the pandemic, it was determined that they paid less attention to protective measures in general. Individuals with high-risk perceptions were significantly more accepting of public health measures to prevent the spread of the disease^(20)^. In a study conducted with medical students in Turkey, it was reported that the students’ risk perception regarding COVID-19 was at a medium level, their protective behaviors were quite high, and risk perception and preventive behaviors had a high positive correlation^(15)^. In addition, in a UK study, researchers emphasized that fear of COVID-19 is “the only indicator of positive behavior change”^(25)^. These findings regarding the COVID-19 pandemic confirm the link between risk perceptions and behavior^(26)^. Studies have shown that individuals who perceive high risk are more compliant with key restraint measures such as staying at home, maintaining social distance, and hand washing^(27)^. A positive correlation was found between anxiety about COVID-19 and self-quarantine behavior in the US, Canada, and Europe^(28)^. In a study in Qatar, risk perception was identified as an important predictor of social distancing behavior^(29)^. A relationship has also been reported between risk perception and intention to perform preventive behaviors, such as frequent hand washing or disinfecting surfaces in young adults in Poland^(30)^. In this study, it has been supposed that the reason for less obedience to protective behaviors over time may be due to moderate risk perceptions, most of the students staying in dormitories, adaptation to the disease, and the characteristics of the Z generation.

This study had some limitations. First, as part of a cross-sectional study, data were collected at a particular time point during the COVID-19 pandemic, which failed to reflect changes in all the variables investigated over time. In our country, midwifery and nursing 4th-grade students were allowed to continue their education during the pandemic period, but the participation rate in the current study was not at the desired level because clinical practices for the relevant courses continued in health institutions and too many scientific studies were conducted by different researchers on this special group. Additionally, since the reflection of students’ risk perceptions on protective behaviors cannot be monitored and their perceptions are evaluated based on self-report, social desirability and reporting bias may be present. In this context, participation in the study and perception assessment based on self-report can be considered as limitations of the study.

## CONCLUSION

This is the important study to explore the impact of midwifery and nursing students’ risk perceptions on their professional commitment. In the two years of the COVID-19 pandemic, the findings showed that the risk perceptions of midwifery and nursing students were moderate, and almost all of the students were vaccinated. It was determined that the students were very compatible with basic measures such as hand washing, wearing a mask and paying attention to social distance to protect from COVID-19 in the first year of the pandemic. The rate of paying attention to social distance, in particular, decreased in the second year. In line with these results, it is thought that healthcare professional candidates’ perceptions of the process, as it is their first pandemic experience, will affect their future professional experiences. In new studies, how the experiences and perceptions of healthcare professionals during the pandemic process affect their current work motivations can be examined through mixed-method studies.

## References

[1] World Health Organization (2023). WHO COVID-19 dashboard [Internet].

[2] Our World in Data (2023). Total COVID-19 vaccine doses administered. [Internet].

[3] Ferrer R, Klein WM (2015). Risk perceptions and health behavior. Curr Opin Psychol.

[4] Lanciano T, Graziano G, Curci A, Costadura S, Monaco A (2020). Risk perceptions and psychological effects during the Italian COVID-19 emergency. Front Psychol.

[5] Caserotti M, Girardi P, Rubaltelli E, Tasso A, Lotto L, Gavaruzzi T (2021). Associations of Covid-19 risk perception with vaccine hesitancy over time for Italian residents. Soc Sci Med.

[6] Patelarou A, Saliaj A, Galanis P, Pulomenaj V, Prifti V, Sopjani I (2022). Predictors of nurses’ intention to accept COVID-19 vaccination: a cross-sectional study in five European countries. J Clin Nurs.

[7] Manning M, Gerolamo AM, Marino MA, Hanson-Zalot ME, Pogorzelska-Maziarz M (2021). COVID-19 vaccination readiness among nurse faculty and student nurses. Nurs Outlook.

[8] Saied SM, Saied EM, Kabbash IA, Abdo SAEF (2021). Vaccine hesitancy: beliefs and barriers associated with COVID-19 vaccination among Egyptian medical students. J Med Virol.

[9] Peres D, Monteiro J, Almeida MA, Ladeira R (2020). Risk perception of COVID-19 among Portuguese healthcare professionals and the general population. J Hosp Infect.

[10] Arslanca T, Fidan C, Daggez M, Dursun P (2021). Knowledge, preventive behaviors and risk perception of the COVID-19 pandemic: a cross-sectional study in Turkish health care workers. PLoS One.

[11] Yıldırım M, Güler A (2022). Factor analysis of the COVID-19 Perceived Risk Scale: a preliminary study. Death Stud.

[12] Brug J, Aro AR, Oenema A, De Zwart O, Richardus JH, Bishop GD (2004). SARS risk perception, knowledge, precautions, and information sources, the Netherlands. Emerg Infect Dis.

[13] Falco A, Girardi D, Dal Corso L, Yıldırım M, Converso D (2021). The perceived risk of being infected at work: An application of the job demands–resources model to workplace safety during the COVID-19 outbreak. PLoS One.

[14] Schneider CR, Dryhurst S, Kerr J, Freeman ALJ, Recchia G, Spiegelhalter D (2021). COVID-19 risk perception: a longitudinal analysis of its predictors and associations with health protective behaviours in the United Kingdom. J Risk Res.

[15] Uzun SU, Çelikyürek NA, Ergin A (2021). Risk perception and preventive behaviors among Turkish medical students during early period of the COVID-19 pandemic. Cukurova Med J.

[16] Taghrir MH, Borazjani R, Shiraly R (2020). COVID-19 and iranian medical students; A survey on their related-knowledge, preventive behaviors and risk perception. Arch Iran Med.

[17] Nehir S, Güngör Tavşanlı N (2021). Med Sci.

[18] Abid A, Shahzad H, Khan HA, Piryani S, Khan AR, Rabbani F (2022). Perceived risk and distress related to COVID-19 in healthcare versus non-healthcare workers of Pakistan: a cross-sectional study. Hum Resour Health.

[19] Ding Y, Du X, Li Q, Zhang M, Zhang Q, Tan X (2020). Risk perception of coronavirus disease 2019 (COVID-19) and its related factors among college students in China during quarantine. PLoS One.

[20] Wise T, Zbozinek TD, Michelini G, Hagan CC, Mobbs D (2020). Changes in risk perception and self-reported protective behaviour during the first week of the COVID-19 pandemic in the United States: COVID-19 risk perception and behavior. R Soc Open Sci.

[21] Yılmaz D, Karaman D, Yılmaz H (2021). Investigation of the effect of intern nursing students’ fear of coronavirus (Covid-19) on anti-vaccine. The Journal of Turkish Family Physician.

[22] Salmon DA, Dudley MZ, Brewer J, Kan L, Gerber JE, Budigan H (2021). Covid-19 vaccination attitudes, values and intentions among United States adults prior to emergency use authorization. Vaccine.

[23] Paul E, Steptoe A, Fancourt D (2021). Attitudes towards vaccines and intention to vaccinate against COVID-19: Implications for public health communications. Lancet Reg Heal – Eur.

[24] Salali GD, Uysal MS (2020). COVID-19 vaccine hesitancy is associated with beliefs on the origin of the novel coronavirus in the UK and Turkey. Psychol Med.

[25] Harper CA, Satchell LP, Fido D, Latzman RD (2021). Functional fear predicts public health compliance in the COVID-19 pandemic. Int J Ment Health Addict.

[26] Lewis A, Duch R (2021). Gender differences in perceived risk of COVID-19. Soc Sci Q.

[27] Dohle S, Wingen T, Schreiber M (2020). Acceptance and adoption of protective measures during the COVID-19 pandemic: the role of trust in politics and trust in science. Soc Psychol Bull.

[28] Nelson BW, Pettitt A, Flannery JE, Allen NB (2020). Rapid assessment of psychological and epidemiological correlates of COVID-19 concern, financial strain, and health-related behavior change in a large online sample. PLoS One.

[29] Abdelrahman M (2022). Personality traits, risk perception, and protective behaviors of arab residents of Qatar during the COVID-19 pandemic. Int J Ment Health Addict.

[30] Sobkow A, Zaleskiewicz T, Petrova D, Garcia-Retamero R, Traczyk J (2020). Worry, risk perception, and controllability predict intentions toward COVID-19 preventive behaviors. Front Psychol.

